# Critical Issues in Scanning Electron Microscope Metrology

**DOI:** 10.6028/jres.099.059

**Published:** 1994

**Authors:** Michael T. Postek

**Affiliations:** National Institute of Standards and Technology, Gaithersburg, MD 20899-0001

**Keywords:** accuracy, backscattered electron, field emission, metrology, scanning electron microscope, secondary electron

## Abstract

During the manufacturing of present-day integrated circuits, certain measurements must be made of the submicrometer structures composing the device with a high degree of repeatability. Optical microscopy, scanning electron microscopy, and the various forms of scanning probe microscopies are major microscopical techniques used for this submicrometer metrology. New techniques applied to scanning electron microscopy have improved some of the limitations of this technique and time will permit even further improvements. This paper reviews the current state of scanning electron microscope (SEM) metrology in light of many of these recent improvements.

## 1. Introduction

During the manufacturing of present-day integrated circuits, certain measurements must be made of the submicrometer structures composing the device with a high degree of repeatability.^1^ These measurements of minimum feature sizes known as critical dimensions (CD) are made in order to ensure proper device operation. For example, the current version of the Intel Pentium^2^ microprocessor operates at 66 MHz; it is reported that by reducing the CD from its current demensions to 0.6 μm the speed of the microprocessor can be increased to 100 MHz or more [32]. The CD and other dimensions must be monitored during manufacture. Optical microscopy, scanning electron microscopy and the various forms of scanning probe microscopies are major microscopical techniques used for this submicrometer metrology. Optical microscopy, undeniably the oldest form of microscopy of the three, has been available for over 300 years. During that time, a substantial amount of maturation has gone into the methodology of optical microscopy. But, even with this time and research devoted to the development of this technique there are limitations to optical submicrometer metrology [72]. These are physical limitations based upon the properties of light Once some of these limitations became recognized it was thought that the scanning electron microscope would then become the metrology tool of choice for sub-micrometer metrology. Unfortunately, limitations also exist using this technique [86, 137]. These limitations are based upon the interaction of the electron beam with the sample. Scanning probe microscopy was then considered the “heir apparent” to the submicrometer metrology “throne.” But, under scrutiny, limitations to this technique were also soon encountered [29,30,41]. These limitations include tip bending during the measurement scan, hysteresis and the need for tip characterization [29, 31]. But, are all these criticisms based upon actual limitations to the tools or are they only limitations to the knowledge of the tool? Are we only looking for a quick answer in the desperation of keeping up with a rapidly moving technology and not looking beyond? Clearly, even with over 300 years of research and development, optical microscopy remains a viable tool in the submicrometer region. Recent modifications and improvements to the optical techniques for near-field microscopy, interference and confocal scanning microscopy, have helped to extend optics further into the submicrometer measurement regime than predicted even 5 years ago. New techniques applied to the field of scanning electron microscopy have improved some of the limitations of this technique and time will permit even further improvements. The field is still open to the scanning probe instruments. Although there may appear to be physical limitations to a particular technique, clever new innovations can help to overcome the shortcomings *once they have been identified* and help to extend the applicability of the technique even further. Eventually, for each of these techniques, an insurmountable wall, based on fundamental physics, must be reached. However, none of these measurement techniques have necessarily reached this wall in the submicrometer measurement region and so all of these techniques continue to have their niche in the measurement of submicrometer structures. In many ways, they are all complementary to each other and each will remain useful for some time to come. In some instances, the strengths of two (or more) of these techniques can be combined to provide a powerful metrology tool. For example, an optical microscope has been combined with an SEM resulting in a dedicated, in-line measurement tool which is designed to accomplish rapid low magnification wafer positioning and pattern recognition optically Then, once the wafer is properly positioned, the instrument automatically switches to the high resolution electron beam for the subsequent measurement. The combination of the SEM with a scanned probe instrument is also possible. Consequently there is no single solution to the submicrometer metrology issue. Similarly, there is no panacea to the measurement of submicrometer structures since one instrument may work better for some applications than others [1]. Each instrument operates on physically different principles and so differences should be expected and anticipated. None of these instruments can be used blindly with the anticipation that good results will happen just because an expensive instrument has been purchased.

This review and a related paper [80] focuses exclusively on current aspects of scanning electron microscope metrology. The state-of-the-art of scanning electron microscope metrology has, in many ways, changed substantially since the topic was reviewed by this author in 1987 [86]. Scanning electron microscopy can still be viewed as a rapidly evolving field in many areas. Unfortunately, this field has also remained somewhat idle in many other ways. It is this contrast that will be reviewed in this paper. But, even as this review is being written, it should be clearly noted that, since this is a very progressive field, new technology is being developed and perhaps employed to improve this instrumentation even further.

## 2. SEM Specifications and Current Capabilities

The SEM is used in a number of applications inside and outside of the wafer fabrication facility (fab). These include: stepper setup [5], stepper lens characterization [129], overlay [109], inspection [55, 124], process control [100], particle analysis [26], as well as, CD metrology [4,28,58,114,119,122, 138]. The SEM is often used as the tool to which all other techniques are compared. Because of the diversity of instrument use, no universal set of specifications satisfying all needs can be defined. Some of the current, *desired* specifications for inline and inspection SEM instrumentation are found in Table 1. This table should be considered to be somewhat generic and not specific to any particular organization. These specifications may be under or over specified depending upon SEM application (production vs development) and demands for a specific facility. This Table is also relatively consistent with specifications established by three major European IC manufacturers in collaboration with the Joint European Submicron Silicon Initiative (JESSI) [37]. The following is a discussion of *some* of the major points of Table 1.

### 2.1 Minimum Feature Size

**Fig. 1:**
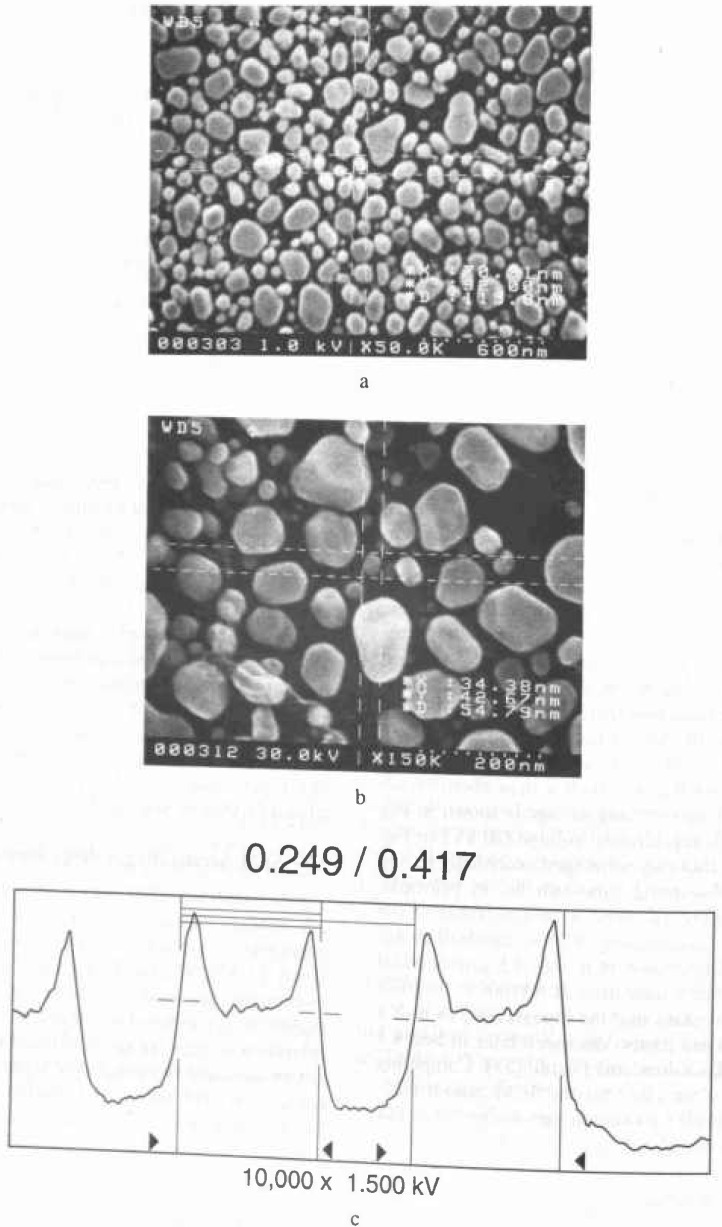
SEM resoluton and measurement capabilities. (a) High magnification, low accelerating voltage (1.0 kV) image showing the measurement of a 70.3 nm gold structure at 50 000× magnification; (b) high magnification high accelerating voltage (30 kV) image showing the measurement of a 34.38 nm gold structure at 150 000× magnification; (c) measurement of a 0.2 μm nominal line (0.249 μm width, 0.417 μm pitch) with the new NIST SRM 2090 prototype sample from the SEM interlaboratory study with a filed emission measurement instrument (courtesy of AMRAY, Inc.).

The minimum feature size generally specified, by most companies, for a comparison such as that found in Table 1 is 0.1 μm. Most fabrication facilities have not achieved this dimension in production. Thus, there is no need to specify for smaller structures. This does not mean that this is the smallest feature measurable by an SEM metrology instrument, *but* the minimum feature predicted tobe fabricated during the life of the specified instrument. The ability of an SEM to view and measure sample structure, as small as or smaller than about 70 nm at low (1 kV) accelerating voltage is shown in Fig. 1a and at high accelerating voltage (30 kV) in Fig. 1b. Anything that can be imaged acceptably having a good signal-to-noise ratio can be, in principle, measured. Figure 1c shows a measurement of the new SEM low accelerating voltage standard prototype SRM 2090. Note that the 0.2 μm nominal linewidth is easily measured at 100 000× magnification. The accuracy and the repeatability of such a measurement are issues discussed later in Sec. 4.1 and also by Larrabee and Postek [53]. Competing technologies often cite that the SEM cannot measure below this 0.1 μm minimum feature size, but this is clearly not the case.

### 2.2 SEM Resolution

The achievable resolution of the SEM has improved substantially over the past 5–10 years. Improvements in electron sources, lenses and electronics have contributed greatly to this advancement, as discussed below. The resolution attainable relates to many factors other than just the instrument capabilities including the composition of the specimen being observed or measured [44,46,78,86]. As shown in Table 2, achievable resolution also depends upon the type and design of the instrumentation being discussed. In recent years, instrument design has gone through a rapid evolution. Generally, an in-line metrology instrument should have 8 nm resolution (or better) at 1 kV accelerating voltage. The European initiative [37] has gone even further and set the goal to be 6 nm at 1 kV accelerating voltage. The determination and maintenance of this performance level is an issue that will be discussed further in Sec. 4.3.2.

### 2.3 SEM Accelerating Voltage Range

Nondestructive SEM operation [64,58,83] generally restricts the metrology instrument accelerating voltage to an arbitrary range from about 0.5 kV to about 2.5 kV. Several dedicated in-line metrology instruments are restricted, by design, to this or a slightly higher range. For special purposes, the accelerating voltage can go higher and if a device will not be damaged or charged the higher accelerating voltage can yield higher signal and instrument resolution. Many laboratory and some on-line SEM instruments routinely operate throughout the accelrating voltage range of 0.5 kV to 30 kV or even 50 kV) What is important is performance in the non-destructive region of the accelerating voltage range for a particular device and not necessarily the specification listed in Table 2, as this can be very instrument and application specific and so the wider range is also listed in Table 2.

### 2.4 Magnification

Metrology, in an SEM, is fundamentally done by identifying two picture elements or pixels in a digitized image and then determining the distance between them. The lateral resolution of the measurement system is fixed by the number of pixels comprising the digital electronics (see Sec. 3.2). Calibration of the SEM magnification effectively determines a known column scan (see Sec. 4.3.1) in both the X and the Y directions. The scan width divided by the number of pixels of the measurement system yields the pixel width or measurement unit. In instruments with a fixed number of pixels (i.e., 512 or 1024), the higher the magnification (relative to the micrograph), the smaller the area on the sample this pixel represents. It is therefore advantageous to make measurements at the highest magnification possible in order to obtain the smallest measurement unit and thus the most sensitive measurement (where the measurement system is concerned). In order for the measurement be meaningful, the pixel size must be less than the required repeatability (Table 1) in order for the instrument to be sensitive to the measurement and that the measure of instrumental repeatability be meaningful [53]. For example, at 50,000× magnification (on a typical micrograph) the pixel width is equal to about 2.25 nm on the sample for a 1024 digital measurement system; twice that size for a 512 digital measurement system. Furthermore, the SEM must also have the resolution, and thus the sensitivity, to detect structural differences at that magnification, or the result is just empty or useless magnification and insensitive measurements (see Sec. 4.3.2).

### 2.5 Measurement Repeatability

The 3 standard deviation or 3*S* repeatability [52, 53] of measurements made with the metrology instrument is generally specified to be at least 1 % or the feature width. This also implies that the feature being measured has a structure variation less than the instrument’s repeatability so that the data is sensitive to instrument repeatability and not the converse [53]. One interesting factor that one must consider when comparing the repeatability of an optical metrology tool to that of the SEM metrology tool is that each instrument is unique in the measurement process. An optical tool can average as much as 1 μm to 2 μm along a line in a single measurement scan depending upon the instrument design. In contrast, a single SEM measurement scan obtains information from as little as only a few tens of nanometers. It would take multiple SEM line scans to average the same sample area. Therefore, any variability of the sample, along the line, is averaged more in the optical measurement than in a typical SEM measurement. Consequently, on the surface it would appear that SEM measurements were less repeatable than optical microscope measurements, but only hecause the SEM measurements were more sensitive to changes in the sample [53] Many factors influence the measurement repeatability of the SEM. A number of these factors have been discussed previously [86] and others are discussed in later sections of this paper. One factor that has *not* been fully explored that might improve measurement repeatability is data oversampling. One difficulty automated measurement systems have is the reproducible determination of edge position. Having more data points available in the proximity of the edge improves the repeatability of the determination of the location of the edge and thus the measurement. The concept of data oversampling was shown to be highly successful in an earlier study on x-ray lithographic masks [92, 93]. Unfortunately obtaining more data may impact throughput which leads to the age old question: “Is, the goal to obtain good data or fast data?”

### 2.6 Throughput

Rapid processing of wafers through an instrument provides a financial advantage to the user. Clearly, one of the main driving forces for the specifications in Table 1 is throughput. Cost-of-ownership modeling [10, 50] has placed a great deal of emphasis on wafer throughput and thus a great deal of engineering effort has been spent on this facility [132]. It must be emphasized that the ideal number of 20 wafers per hour listed in the Table depends greatly upon the sampling plan employed. It should also be noted that an instrument with high throughput but poor overall resolution or measurement sensitivity provides no advantage at all.

### 2.7 Availability and Mean Time to Failure

Availability or uptime of a metrology tool for most production fabs is required to be greater than 95 %. This should also be expected for any modern laboratory instrument, not just those in the wafer fabs. If the instrument is unavailable due to a failure, maintenance, or lack of availability of parts, the instrument is considered to be down and unavailable for use by some definitions. If an instrument cannot do its assigned job function, money is lost since the production line is delayed. Similarly, in a laboratory situation a down instrument may cost the facility money due to delayed work or lost revenue from canceled laboratory appointments, providing embarrassment to both the user and the instrument manufacturer or service organization. Such a broken instrument or a “hard down” situation (e.g., a filament failure) is obvious and easily determined. But what about a subtle down condition when the instrument is apparently functioning normally but the measurement data generated is marginal because of a resolution, or sensitivity loss? How and at what frequency is this checked? More on this topic is presented in Sec. 4.3.2.

### 2.8 Particles

Particle metrology and characterization is now becoming a growing field. Particles are a bane of semiconductor processing [3, 74]. The SEM has numerous moving parts. Each can generate particles through wear mechanisms. As the wafer is transferred into and out of the system, particles can be generated from contact with the wafer transfer machinery. Movement of the wafer into and out of the vacuum causes some degree of turbulence which can mobilize particles possibly depositing them on the wafer surface. Particles can also be formed by temperature and pressure changes during the sample exchange process leading to water vapor condensation, droplet formation and liquid-phase chemical reactions [52]. Modern SEM instrument design minimizes particle generation [74] Specifications found in Table 1 indicate that the inspection instrumentation should induce fewer than two particles per wafer pass. Clearly, the size of the wafer, as well as the size of the particles, must also be considered in such a specification in order to make it meaningful to a specific process. Reduction of particle generation is also important to the performance of the instrument since a charged particle landing on a sensitive portion of the instrument can rapidly compromise the resolution of the SEM, especially at low accelerating voltages.

### 2.9 Measurement Scan Linearity

Historically, the SEM does not necessarily do flat field scanning [107]. It is imperative that any measurements made with this instrument be made in the center of the scan field. It is also imperative that little or no scan shift be used (unless fully tested) for the same reason. This ensures that the measurement is done in the most linear part of the scan. Desired European specification indicates a scan linearity of 10 nm (3*S*) as measured on 7 points on the SEM monitor [37]. However, it should be clearly noted that it is not the display monitor scan linearity that metrologists should be concerned with, but the measurement scan linearity.

## 3. Instrumentation Improvements

The scanning electron microscope metrology instrument has undergone a number of design improvements during the past few years. Many of these improvements have been generally applicable across the board in the field of scanning electron microscopy and some of them have been specific to semiconductor processing applications. Improvements in: electron sources, digital imaging, lens designs and electron detectors are four areas where fundamental design improvements have been instrumental in improving submicrometer SEM metrology, as well as the entire field of scanning electron microscopy.

### 3.1 Improved Electron Sources

In 1987, when the first review of the topic was done by the author [86], the predominant electron sources were the thermionic emission type cathodes, especially tungsten and lanthanum hexaboride (LaB_6_). Lanthanum hexaboride filaments became more prevalent for low accelerating voltage applications because of the increased brightness and decreased source diameter in comparison to tungsten filaments (Table 3). Cerium hexaboride (CeB_6_) is a new innovation which is similar in performance to the lanthanum hexaboride filament [12]. Point-cathode electron sources or field emission instrumentation were available for semiconductor processing, but the concept was still in its infancy at the time of the earlier review and few commercial instruments were available with that capability. Today, a wide variety of both laboratorytype and in-process type instruments are commonly available with field emission technology. For most in-line semiconductor processing applications, only the field emission instruments provide the high resolution necessary for this type of work, especially at the low accelerating voltages needed for nondestructive inspections (Table 2). In the near future other electron sources, such as nanomeler-sized field emission tips, may also become available [96,97,120,121].

#### 3.1.1 Point-Cathode Electron Source Types

There are two basic categories of point-cathode electron source types used in the current SEM metrology instruments: Cold cathode Field Emission (CFE) and Thermally-assisted Field Emission cathodes (TFE). Although the concept of field emission can be traced to the early work of Wood [150] and was used in early instrumentation by Zworykin et al. [152], it was not until the late 1960s that Crewe and his coworkers [15] developed a successful cold cathode field emission source that was later introduced into commercial instrumentation [145,146]. CFE has had a relatively long history in scanning electron microscopy and SEM metrology and was the first type of field emission cathode to be applied to semiconductor processing instrumentation. Thus, CFE dominates the field by sheer numbers of instruments.

For many applications, such as analytical microscopy and microfabrication, the CFE was not capable of producing the high currents and large spot sizes needed [59,75]. Work to develop a high current thermally assisted field emission cathode with relaxed vacuum and environment requirements was then begun [127,128,133,147]. There have been several designs of thermally assisted field emission cathodes developed. The two major types are: the Tungsten <100> built-up Emitter (TE) and the ZrO/W <100> cathode Shottky Emitter (SE). At the current time, the ZrO/W is the more commonly used of the two types of thermally assisted field emission source in modern laboratory and SEM-based metrology instruments.

Instruments utilizing either CFE or SE currently populate the SEM metrology field. Each type has its advantages and disadvantages. It is up to the informed user to test and to determine the type of source that suits the application. The characteristics of the various electron sources, as they are currently understood, including CFE and SE, are summarized in Table 3 and are briefly discussed below.

##### Field Emission Cathodes

**Fig. 2:**
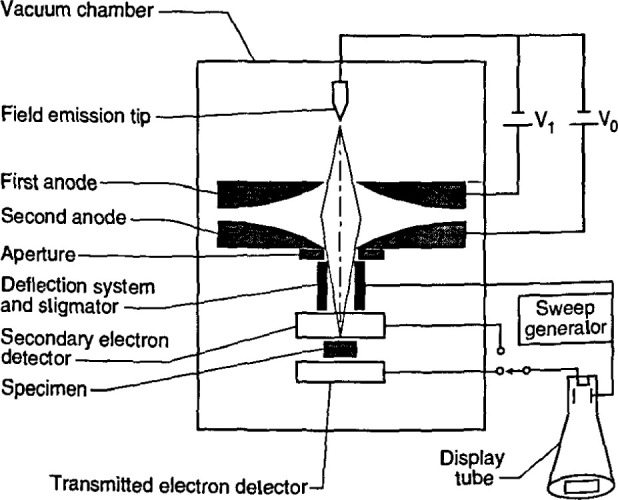
Cold field emission electron microscope column of the design of Crewe et al. (redrawn from Crewe el al., 1969).

Cold field emission cathodes, developed for use in the scanning electron microscope by Crewe and co-workers [15], have an advantage of providing a relatively high-current electron probe having a low energy spread, high brightness, and a small virtual source diameter, especially at low acceleratine voltages. The CFE source diameter is sufficiently small that the electron gun alone (as shown in Fig. 2) without any additional condenser lenses is capable of producing a 10 nm probe [17, 18] From Table 2 it can be observed that depending upon the type of instrument design, better than 1 nm resolution may be reached with an instrument equipped with a field emission electron source. The overall advantages afforded by CFE are offset somewhat by the rigorous requirements for ultra-high gun vacuum (Table 3) and some fluctuation (flicker) in the emission current. The emission current fluctuation is readily compensated for by constant beam monitoring and feedback control [13,115,116], and also (with the newer instruments) through digital frame averaging, and in general, is not an issue of concern.

##### Schottky Emission

**Fig. 3:**
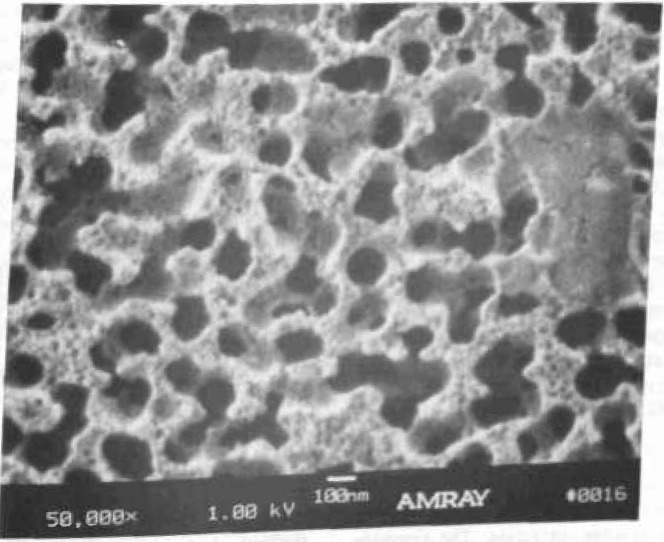
High resolution low accelerating voltage imaging using a thermally assisted field emission electron source (courtesy of AMRAY, Inc.).

The second basic category of point-cathode electron sources is the thermally assisted field emission cathode. In this mode of operation, the cathode is heated and thus vacuum requirements are reduced and the emission current is relatively stable [134]. Because of its lower work function, the use of the Schottky point emitter (SE), such as the Zirconiated/tungsten <100> (ZrO/W) point cathode [134], is preferred. This source can produce a high current electron beam with a slightly poorer energy spread. This differs from cold field emission by an amount as small as about 8 % to 10 % depending upon how evaluation criteria are established [134, 136]. Since this source is currently being used for a number of different applications, the operational characteristics and parameters of the source are quite varied. Thus it is quite difficult to tabulate a direct comparison of source characteristics. For SEM metrology applications the SE source is generally operated with conditions resulting in the lowest energy spread (0.3 eV) possible for that type of source. Under these conditions using test samples, comparable resolution (as related to image sharpness as discussed in Sec. 4.3.2) to similarly equipped CFE instruments has been obtained (Fig. 3). Unlike the CFE, the larger source diameter characteristic of this type of electron source requires the use of an extra condenser lens in the electron microscope column in order to increase the source demagnification. The need for increased demagnification also provides a positive secondary effect since it also results in increased demagnification of external noise such as vibration and fields affecting the source.

### 3.2 Improved Digital Image Storage and Image Analysis

Another of the major advancements applied to SEM metrology during the past few years has been the incorporation of digital imaging technology. Advancements in semiconductor technology, notably the availability of less expensive, high-density memory chips and the development of inexpensive high speed analog-to-digital converters, mass storage, and high performance central processing units, have fostered this revolution. Today, most modern SEM metrology instruments have digital electronics as a standard feature. These instruments generally have 8 bit or 256 gray levels, with *at least*, 512 pixel by 512 pixel density operating at TV rate. Many of the more modern metrology instruments operate at either 1024 by 1024 or higher pixel density and at least 10 bit or higher gray levels [87]. In addition, current slow-scan commercial frame-grabber cards, directly applicable to the SEM, can have upwards of 12 bit to 14 bit lateral resolution, which permits image acquisition and measurement at 4096 by 4096 resolution or greater [87]. Pre-digital electronics metrology SEMs were plagued by the problem of having a poor signal-to-noise ratio, especially at low accelerating voltages and TV scan rates. Recent developments in field emission filament technology improved that situation, but parallel development of the modern digital imaging technology brought both of these technologies together into an extremely powerful tool with exceptional flexibility. Some of the advantages afforded by digital imaging include:

#### 3.2.1 Pattern Recognition

Rapid transfer of the wafers within an in-line instrument requires a rapid, accurate, pattern recognition system for high throughput. Depending upon the system design, the pattern recognition process can be accomplished either with an optical system, the electron beam system, or both in conjunction. In actual use, the probability of detection is often highly substrate dependent. For current metrology instruments, this leads to one of the major causes of measurement variability (Table 1).

#### 3.2.2 TV Rate Scanning

TV rate scanning is not new to SEM, but previously this type of operation had to be done at increased beam currents and thus reduced resolution in many instruments. Today, essentially the “slow scan” presentation of the SEM is gone and is replaced with a flicker-free, real-time TV image. Digital integration of poorer signal-to-noise images is transparently accomplished by frame buffering and frame averaging of the video signal. TV rate scanning has also been shown to be useful in the reduction of charging on many samples [146].

#### 3.2.3 Digital Image Storage

Image archiving of the digital images, either to floppy disk or hard disk, provides a permanent record that is inexpensive and easy to retrieve. Image quality is identical to the originally stored image. Standardized file storage such as the TIFF (or other) file format can enable importation of the images into desk-top computers, particularly statistical analysis and word processing programs (see Sec. 4.7).

#### 3.2.4 Paperless Image Transmission

The image and measurement data can be transmitted via data lines to remote locations. It is conceptually possible to view the SEM image from a remote location and actually operate the SEM from that location in real-time. Today, the production engineer does not have to be suited-up in the wafer fabrication facility in front of the instrument to view the wafers or measurement results, or for that matter to operate the instrument.

#### 3.2.5 Real-Time Image Analysis/Processing

Digital enhancement of the image can be done transparently, as the image is acquired, and the image and data can be processed at the SEM console. It should be noted that in many laboratory and metrology instruments the signal undergoes some processing as it is transported through the video chain. The operator may not even be provided with the ability to view the “raw” data. Blindly allowing the image or data to be processed should be approached with caution and raw data should always be able to be obtained from a metrological instrument.

#### 3.2.6 Optimization of Operating Conditions

Digital SEMs can automatically optimize the operating conditions, such as the brightness, contrast focus, and astigmatism correction. The operator can save optimum operating conditions for a particular sample class, then reload them as needed. Many of the instrument parameters that need to be changed when instrument conditions are altered can be changed automatically through look-up tables.

Until a few years ago, digital imaging was severely limited by the power, memory, and cost of the computer systems available, and, therefore, much of the digital imaging was done externally through interfacing to a powerful minicomputer coupled to an x-ray microanalysis system. Today, many desktop computers have computing capabilities surpassing these early minicomputers. Computer systems are now small and inexpensive enough to be directly incorporated into the SEM electronics console as a standard component by the SEM manufacturer. This concept presents a major advantage because the digital architecture of modern SEMs now permits the application of a whole host of peripheral technologies associated with, and being developed for, the personal computer industry to be readily applied to the SEM and SEM metrology.

### 3.3 Improved Lens Design

The semiconductor wafer samples being viewed in the scanning electron microscope metrology and inspection instruments are by their nature quite large. Instruments are being designed to accommodate up to 200 mm diameter and larger wafers. Moving samples of such large dimensions rapidly within the specimen chamber, in vacuum, has been a difficult engineering problem. Not only did specimen chambers and stages need to be increased in size and travel, but also final lens technology required improvement.

**Fig. 4:**
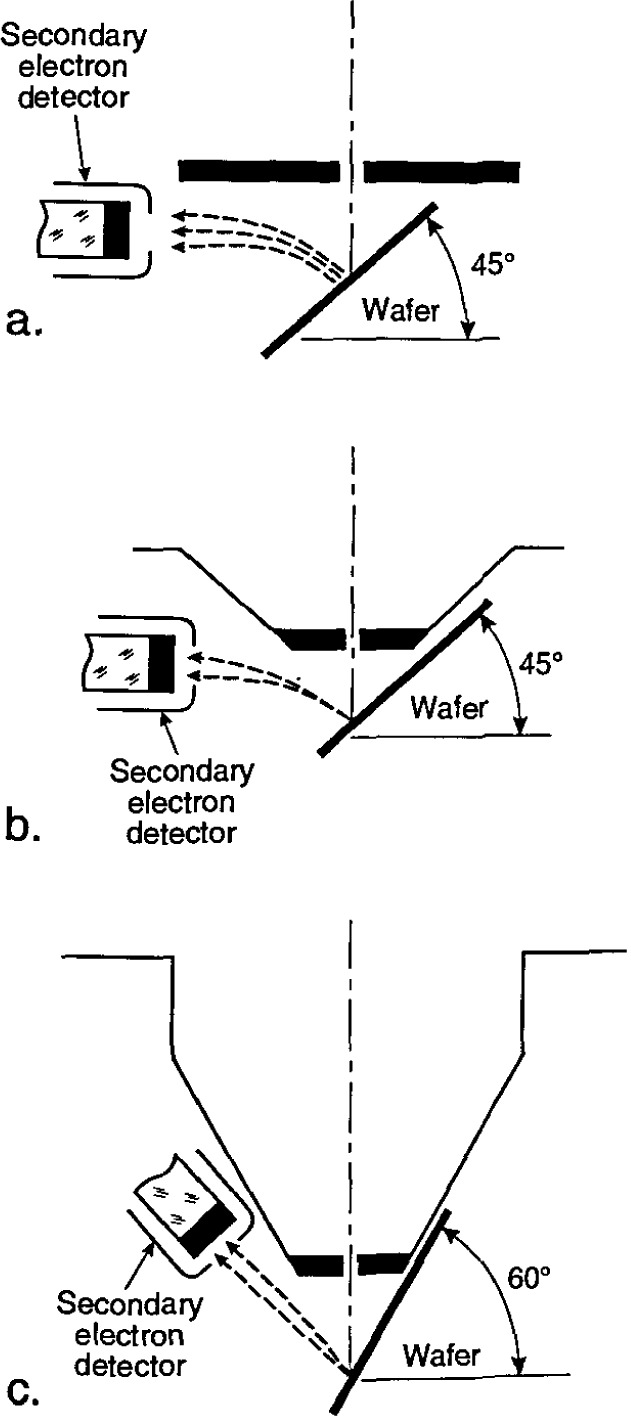
Drawing of the comparison between different types of “pinhole” tenses. (a) Early version flat lens; (b) 45° conical lens; (c) 60° conicat lens.

At the time of the earlier review [86], flat “pinhole” lens technology predominated (Fig. 4a). This was the state-of-the art of the instrumentation at that time. Later, 45° and 60° conical lens technology with improved low-accelerating voltage performance began to improve the manipulation and viewing of the wafer within the specimen chamber (Fig. 4b and Fig. 4c). However, these were still pinhole-type lenses and limitations imposed by the sample/lens geometry on the instrument resolution remained. For example, even a 60° conical lens having a broad front face would still be restricted to rather long working distances with highly tilted samples. Two improvements in lens design directly applicable to the in-line wafer instrumentation were introduced. The first improvement was through-the-lens electron detection and the second was extended-field lens technology.

#### 3.3.1 Through-The-Lens Electron Detection

**Fig. 5:**
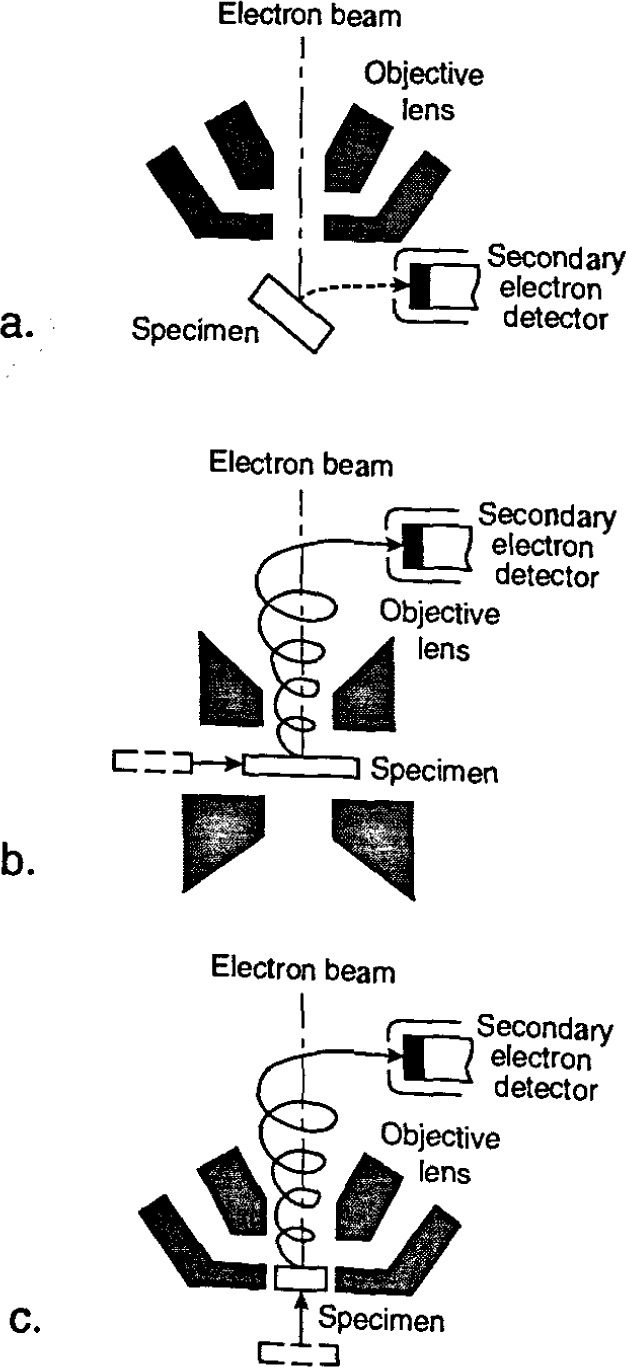
Comparison of SEM final lens design. (a) Conventional flat lens technology; (b) in-lens SEM technology showing the detector above the lens; (c) conventional SEM with in-lens capability.

**Fig. 6:**
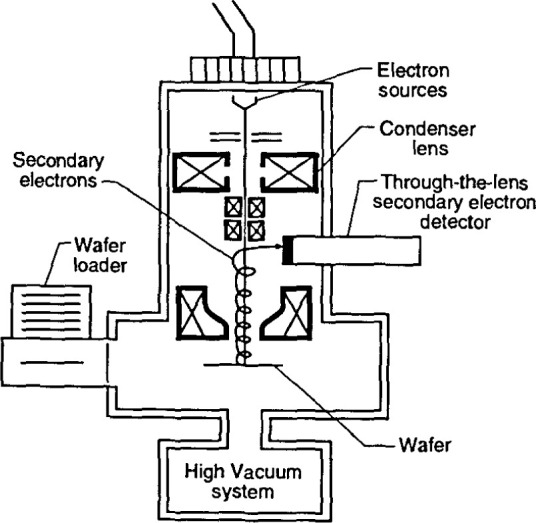
Drawing of a typical metrological SEM with “through-the-lens” electron detection (redrawn after Hitachi).

The term “through-the-lens detection” relates to the fact that signal electrons are transported back through the lens that focused the primary electron beam on the sample; this concept has been reviewed by Kruit [49]. Pinhole lenses have always been restricted in that the space between the lens and the sample had to be shared by the electron detector (Fig. 5a). Therefore, in typical SEM application, some open working distance between the final lens and the sample surface is required to permit electron collection. Scanning transmission electron microscopes have, for many years, been able to place specimens directly into the bore of the objective lens, effectively immersing the sample into the lens field at essentially very short working distances. Unfortunately, the space in the lens is quite small and restricts the size of the specimen to be viewed to a few millimeters. The immersion lens concept and the through-the-lens electron collection technique was adapted into ultra-high resolution scanning electron microscopes (Fig. 5h). In this configuration, secondary electrons were caught in the field of the lens and drawn upward to be collected by the detector placed above the lens. However, the sample size restriction remained. Opening up the bore of the final lens and placement of the electron detector into the space above the lens also improved this geometry for normal SEM operation. In some instances, a small sample could even be carefully raised into the final lens bore for higher resolution (Fig. 5c). These solutions allowed shorter working distances, even for larger samples, and thus higher source demagnification and attainable instrument resolution. This approach proved to be very successful for in-line wafer metrology instruments since there is no need for sample tilting; thus the wafers could be viewed at short working distance with high resolution and signal collection. Many in-line metrology instruments arc based upon this concept (Fig. 6).

#### 3.3.2 Extended-Field Lens Technology

**Fig. 7:**
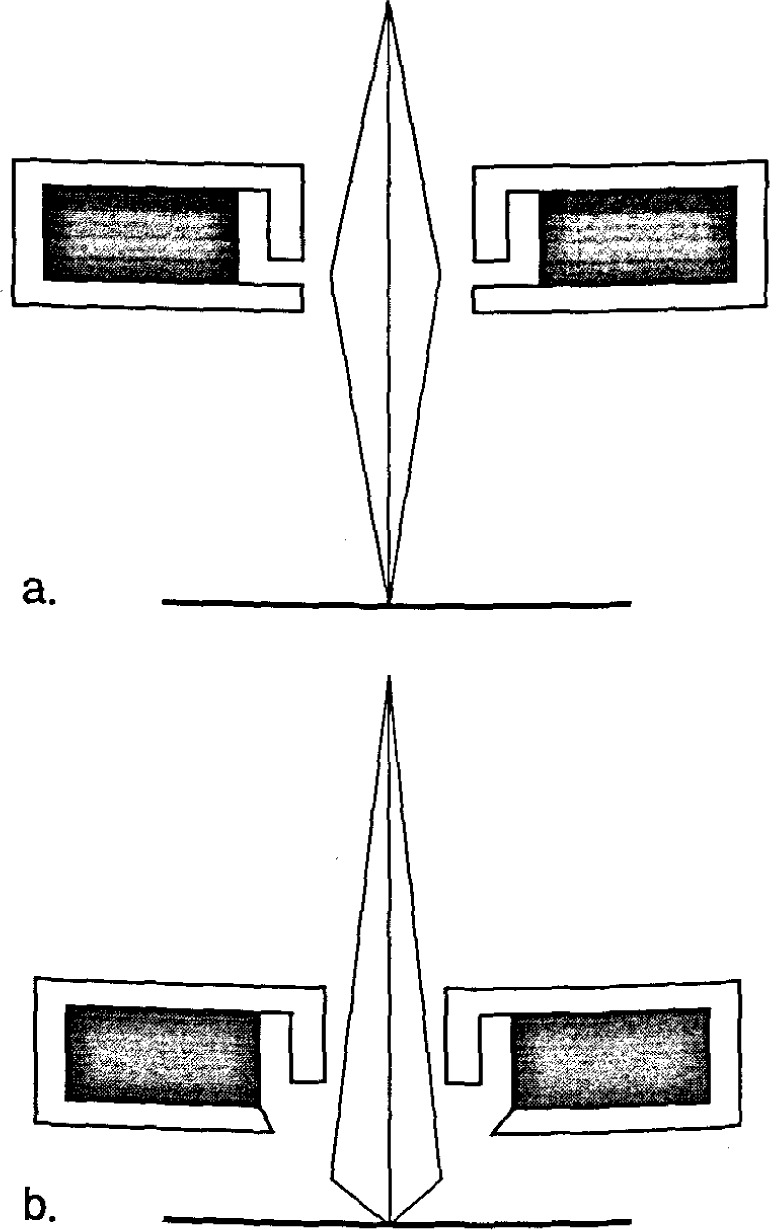
Extended-field lens technology. (a) Standard lens technology; (b) extended-field lens technology where the focusing field is extended beyond the bulk of the lens, thus permitting short working distances with large samples.

**Fig. 8:**
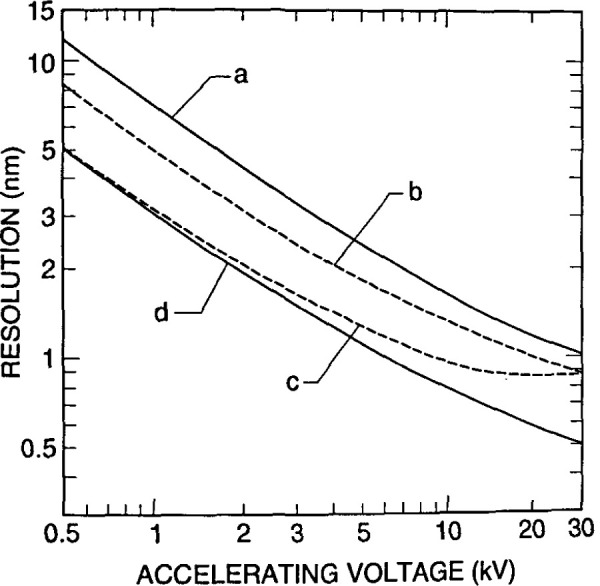
Graphical comparison of the calculated resolution capabilities of different designs and applications of field emission scanning electron microscopes. (a) Standard “pinhole” type final lens with the secondary electron detector in the “normal” location within the specimen chamber; (b) extended-field lens with the electron detector in the “normal” location; (c) extended-field lens with the detector positioned above the lens; (d) in-lens ultra-high resolution instrument (courtesy of Hitachi Scientific Instruments).

**Fig. 9:**
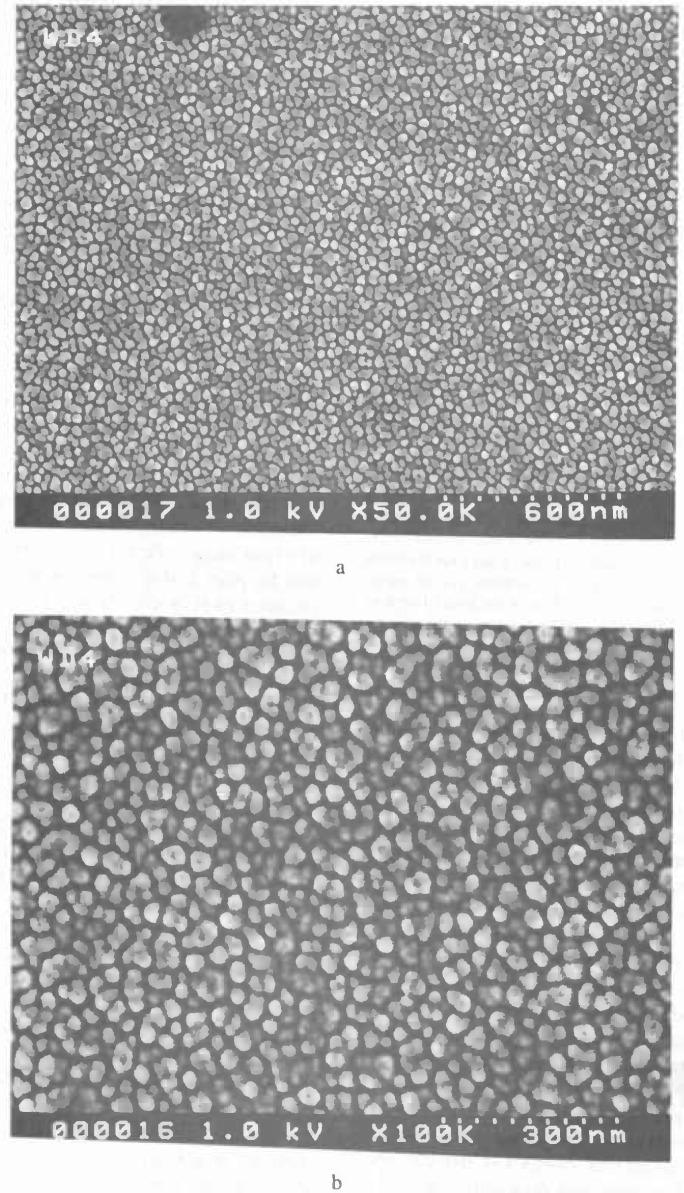
Low accelerating voltage (1.0 kV), high resolution image using an instrument with extended field technology the sample is “grass” on a silicon wafer, (a) Lower magnification image taken at 50 000 × and (b) higher magnification image taken at 100 000 ×. (Courtesy of Hitachi Scientific Instruments.)

It is well known that to obtain the highest resolution in scanning electron microscopy, the shortest working distances are required. Placing the sample into he bore of the final lens near the principal plane of the lens is another alternative (as discussed above), hut such an approach is limited to very small sample. Mulvey [67] proposed the design of a new type of lens, referred to as a snorkel lens, where the imaging magnetic field of the lens extends entirely beyond the lens structure. In essence, rather than placing the specimen into the bore of the lens, the lens extends the field toward the sample. Employing an inverted snorkel or extended-field type lens as the final lens of the scaning electron microscope enables a large sample to he essentially immersed in the field of the lens external to the bulk of the lens (Fig. 7). Because of the very short working distance resulting from this lens concept, high resolution is possible, especially if through-the-lens electron collection is also employed (Table 2). For laboratory and inspection instruments, a secondary electron detector could be placed in the sample chamber as in the conventional design (Fig. 5a) and also placed above the lens for extremely short working distance, high resolution operation (Fig. 5c). Either detector could be used depending upon the need. Fig. 8, shows a graphical comparison of a field emission laboratory-type instrument with “pinhole” lens technology to one having extended field lens technology (with two detectors – upper and lower – as described previously). Apparent in the graph is the substantial improvement in resolution possible with this technology, which approaches that of the ultrahigh resolution in-lens instrument. Effectively, instruments with this technology can now resolve as well at low accelerating voltage (1.0 kV) as instruments equipped with lanthanum hexaboride can resolve at high accelerating voltage (Fig. 9).

### 3.4 Improved Electron Detection Capabilities

**Fig. 10:**
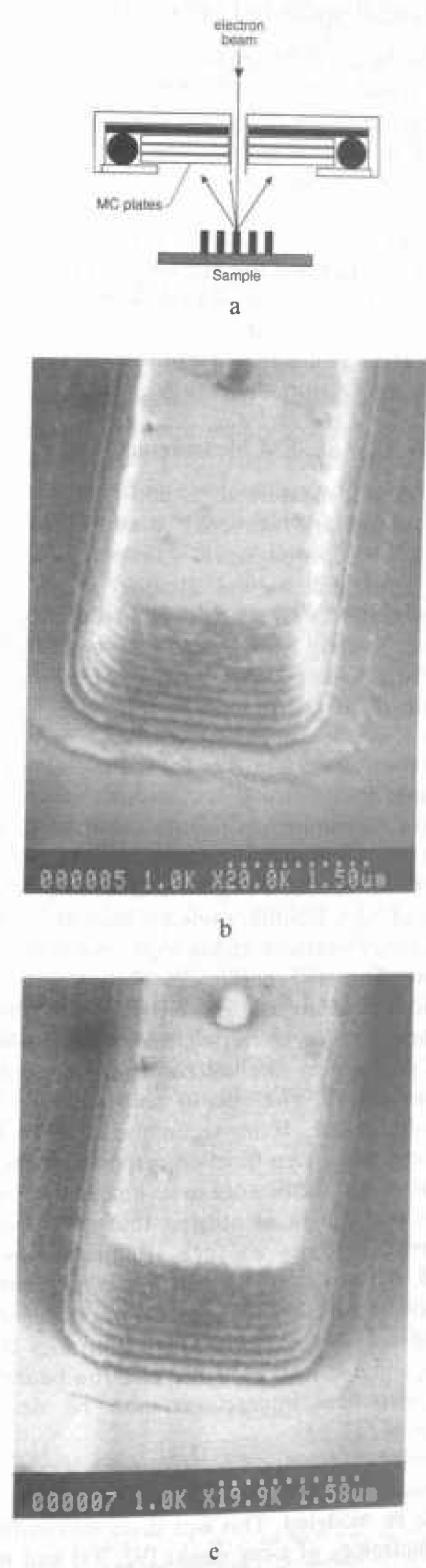
Microchannel-plate electron detector. (a) Drawing of the detector showing its coaxial placement above the sample; (b) secondary electron image; (c) backscattered electron image.

At the time of the first review of SEM metrology [86], most of the scanning electron microscopes used the common Everhart/Thornley (E/T) detector [25], or a variation, as the main detection system for secondary electron imaging. The original detector had a positively biased grid for the collection of secondary electrons, and this design has served well for over 25 years for general purpose SEM operation. Unfortunately, this detector design is generally quite large and intrusive in the specimen chamber (Figs. 4 and 5). Furthermore, the varied applications of the modern scanning electron microscope have, in many ways, been expanded beyond the capabilities of this detector system, especially for low accelerating voltage studies and for SEM metrology. When the picoampere beam currents characteristic of nondestructive, low accelerating voltages are used, the performance of the E/T detector degrades and yields a poor signal-to-noise ratio. The E/T detector also suffers from alignment difficulties, often because of its non-coaxial mounting position with respect to the sample and the electron beam, or the uneven distribution of the collection field. It is imperative to metrology that the signal being measured be symmetric. Asymmetric signal collection is especially troublesome where linewidth measurements of microcircuit patterns are being made [73, 113]. These limitations, and others, have led recent investigators to reconsider secondary electron collection mechanisms and detectors. In order to improve the electron collection geometry, Volbert and Reimer [140] and Suganuma [123] proposed using two opposed E/T detectors to improve signal collection efficiency and symmetry. Other workers have placed the electron detector on-axis with the electron beam in the tilt plane in an effort to improve collection symmetry [73]. Schmid and Brunner [117] developed a quadruple electron detector for use as a high efficiency electron detector for low accelerating voltages. Other workers [88,89,112,113,114] proposed using microchannelplate (MCP) type detectors (Fig. 10a) and this detector proved to be quite successful. Since that time, MCP detectors have been used extensively in many SEM metrology applications. As shown in Figs. 10b and 10c, these detectors can be used to collect the “secondary” electron image or the backscattered electron image (see Sec. 3.4.1). The MCP detector can be placed above the sample in the specimen chamber or even into the microscope column as an in-lens detector [66].

#### 3.4.1 Backscattered Electron Detection Technology, Collection, and Measurement

The “secondary” electron signal, usually collected and measured in the SEM, is composed not only of those secondary electrons generated from initial interaction of the electron beam as it enters the sample (SE-1), but also of secondary electrons generated by the escape of elastically and inelastically scattered electrons when they leave the sample surface (SE-2 and SE-3). The emitted backscattered electrons (BSE) can interact singly or multiply with other structures on the sample or other internal instrument components and generate more secondary electrons; they can also be collected as a component of the secondary electron image if their trajectory falls within the solid angle of collection of the electron detector [22, 79].

A fourth contribution of secondary electrons (SE-4) to the signal results from the interaction of the primary electron beam with internal column components (i.e., apertures). The magnitude of the SE-4 contribution is generally small and is instrument specific. It has been calculated that the number of remotely generated electrons (i.e., energy less than 50 eV) is much larger than those generated from the primary electron beam interaction by a factor greater than three [118]. Peters [79] has measured the components of the secondary electron signal from gold eryslals and has found that, depending upon the sample viewed, the contribution of the SE-2 is approximately 30 % and the contribution to the image of the SE-3 electrons is approximately 60 *%* as compared to the approximately 10 % of the image contributed by the SE-1 derived signal. The standard Everhart/Thornley type secondary electron detector does not discriminate between these variously generated electrons and thus the collected and measured secondary electron signal is composed of a combination of all these signal forming mechanisms. The difficulties in interpreting this composite signal can lead to measurement errors that can be highly variable and that have a strong dependence upon sample composition, sample geometry, and to a lesser or greater extent (depending on instrument design), upon other physical factors such as an instrument’s internal geometry that induces anomalies in the detector collection field (i.e., stage motion). Furthermore, since this signal is highly variable and often instrument specific, it is extremely difficult to model.

Relative to the pure backscattered electrons, early workers with the SEM were concerned mainly with imaging and not metrology. Metrological applications often require a different way of thinking and operation. These workers considered all the signal derived from the backscattered electrons to be low in resolution, generally providing only atomic number information and background to the image. Wells [148, 149], using the low-loss method for several classes of materials (including photoresist), demonstrated that this concept was inaccurate, and that high-resolution imaging of backscattered electrons could be done under specific conditions. The behavior of the backscattered electrons has also been modeled by Murata [68, 69], who showed that there is a predominant component of the backscattered signal that is unseattered and high in energy and is therefore believed to carry high resolution information. The high resolution potential of the backscattered electron signal was also later experimentally demonstrated using the converted backscattered secondary eleclron (CBSE) technique at high accelerating voltages [65,66,101,141]. Later, using field emission instrumentation at low accelerating voltages, the CBSE technique was used successfully by Postek et al. [91] to obtain high resolution images, low accelerating voltage backscattered electron images of uncoated photoresist (and other samples). The concept of the high resolution nature of the backscattered electron image is further supported by the work of Joy [45] who demonstrated that the relative nature of the secondary electrons and backscattered electrons can be inverted at low accelerating voltages. In low atomic weight samples such as photoresist or polyethylene, the depth of information represented in the backscattered electron image is about 0.3–0.5 times the electron range [43], while the secondary electron image corresponds to about three times the mean secondary electron escape depth [45]. This results in a potential loss of surface detail in the secondary electron image, due to the longer escape length of the secondary electrons at low accelerating voltages relative to that of the backscattered electrons. This can also result in measurement vari ability. Differences between measurements using the backscattered electron signal and the secondary electron signal have also been demonstrated [87, 90]. In one instance, on a nominal 2.5 μm silicide on silicon line at 30 keV accelerating voltage, measurement broadening associated with a width measurement of the standard secondary electron signal was shown to be 0.2 μm larger than the backscattered electron signal derived from the same sample under similar conditions. It was also demonstrated, in that same study, that under the experimental conditions chosen, the measured backscattered electron signal was less prone to random variations, thus improving its measurement repeatability compared to the secondary electrons. With the microchannel-plate electron detector, Postek [81] demonstrated that backscattered electrons derived from a low accelerating voltage electron beam could be collected and measured. Comparison measurements of the secondary and the backscattered electron images using the same MCP detector showed results similar to the earlier study [90]. Again, the measured values of the structures using the backscattered electron signal were smaller and had less variability. The backscattered electron signal did not demonstrate the measurement broadening effect shown by the collection of the secondary electrons. Backscattered electron measurement capabilities have been recently been adopted in in-line metrology instruments for linewidth measurement and the measurement of contact holes [66]. To date, the use of the backscattered electron signal has yet to be fully implemented in SEM metrological applications largely because of the weak signal generated at low accelerating voltages. However, the distinct advantage presented by this mode of operation, in contrast to the “secondary” electron detection mode, is its ability to be readily modeled, thus providing the potential for accurate metrology (see Sec. 4.1.1).

## 4. Areas Requiring Further Improvement

The SEM has evolved from an instrument used mainly to make micrographs of interesting samples with high resolution and depth of field to a metrology tool in a period of less than 10 years. During this time, many areas have been improved; however, others still require work. These problems are not insurmountable obstacles, but do require attention in order to bring the SEM to its full potential as an accurate metrological tool. As with the previous improvements found in Sec. 3, attention to many of these problems will improve the entire field of scanning electron microscopy.

### 4.1 Accuracy of SEM Measurements

Accuracy of measurements and repeatability of measurements are two separate and distinct concepts [53]. Process engineers want accurate dimensional measurements, but accuracy is an elusive concept that everyone would like to deal with by simply calibrating their measurement system using a standard developed and certified at the National Institute of Standards and Technology. Unfortunately, it is not always easy for NIST to calibrate submicrometer standards or for the engineer to use standards in calibrating instruments. Accurate feature-size measurements require accurate determination of the position of *both* the left and right edges of the feature being measured. The determination of edge location presents difficulties for all current measurement technologies because of the reasons discussed earlier in this paper. Since linewidth measurement is a left-edge-to-right-edge measurement (or converse), an error in absolute edge position in the microscopic image of an amount ΔL will give rise to an additive error in linewidth of 2ΔL. If any technique could be found that produces a step-function response at the location of the geometric edge in its image, there would be no problem in identifying that edge position. However, to date, no such technique has been found. *Without an ability to know with certainty the location of the edges, measurement accuracy cannot be claimed.* For accurate SEM metrology to take place, suitable models of the electron beam/specimen/instrument interactions must be developed and used [52, 53].

In order to develop suitable models it may also be necessary to modify the SEM design to make it easier to be modeled. This was done successfully for the metrology of x-ray masks [92, 93] and may be equally successful for the backscattered electron image (see below).

#### 4.1.1 Improved Electron Beam Modeling

Using current SEM design philosophy, meaningful electron beam modeling is very complicated to do on the current SEM designs. This is because numerous factors contribute to the derivation of the image and thus to the model. It is necessary to model not only the electron beam/specimen interaction, but also the contribution of the specimen chamber, detector geometry, detector sensitivity, electron collection fields, amplification bandwidth, as well as other factors. A great deal of fundamental information needs to be known about each particular instrument. The electron beam model must also take into account the influence on the measurement posed by the proximity to other structures or underlying layers [107, 108]. Proximity effects are well recognized in electron beam lithography and they must be equally recognized as a complication to electron beam metrology. Isolated lines present a different linescan from those in a nested array. Modeling will help us to understand this phenomenon. Electron beam modeling is currently an area of active interest for metrology and other SEM applications.

The most common approach to electron beam modeling has been to use the Monte Carlo technique [19,40,42,51,70], although other approaches have been considered [71, 142]. These other approaches include the use of transport equation theory [103] and the use of a cylindrical envelope model [33]. Electron beam modeling has been done on both the secondary and the backscattered electron images [34,42,56,57,71,86,110]. Unfortunately, since there are so many contributions to the normal secondary and even the broadband backscattered electron image, it is very difficult to isolate individual contributions. Work using the transmitted electron detection (TED) mode on a unique sample, such as the mask used for x-ray lithography, demonstrated that by restricting the contributing factors, a great deal of information could be obtained from the theoretical and the experimental data [92, 93]. Using the transmitted electron image, a relatively rapidly changing intensity in the vicinity of the true edge position is identifiable. It can, therefore, be made inherently less sensitive than the conventional secondary electron based SEM modes to this source of error in linewidth measurements. The TED technique is not inherently more or less accurate than other SEM modes for pitch measurements because pitch measurements are not subject to this type of error as long as the two lines in question have similarly shaped left and right edges.

##### Lithography Masks as a Model System for the Development of other Accurate SEM Standards

**Fig. 11:**
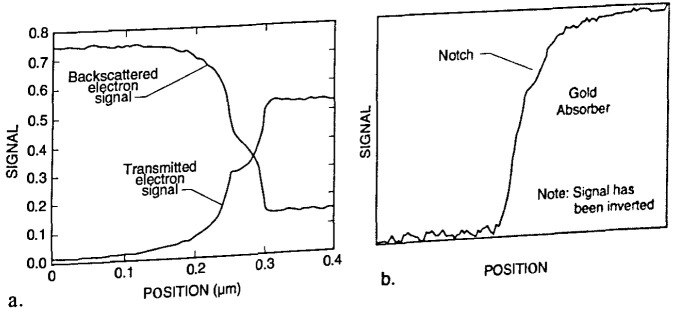
X-ray mask metrology. (a) Monte Carlo modeled data of the transmitted electron signal and the backscallered electron signal showing the appearance of the characteristic notch in both modes of electron collection; (b) digilized field emission SEM micrograph of the transmitted electron signal demonstraling the presence of the nolch in the experimental data.

The x-ray lithography mask provides a unique sample for the development of future accurate dimensional SEM standards. Accurate electron beam modeling has been developed for transmission electron detection for this type of sample and accurate measurements have been made [92, 93]. The development of the model for the transmitted electrons also encompassed the simultaneous development of a model for the backscattered electrons. During this research, it was predicted by the model that both the transmitted electron signal and the backscattered electron signal contained important information about specimen characteristics, especially edge location and wall angle (Fig. 11a). These predictions were confirmed experimentally (Fig. 11b). Comparison work between experimentally obtained data and the computed data of both the TED and the BSE images is currently underway at NIST and continued model development to include the secondary electron image is also planned. Further, comparison of the experimental and theoretical data relative to portions of the x-ray mask not etched to the thin support membrane will also be instructive and is in progress.

### 4.2 SEM Measurement of Depth, Height and Wall Angle

One of the common criticisms of the scanning electron microscope is the perceived inability of the SEM to provide depth measurement. This is a misconception based upon the lack of full development of this facility for general use, as well as metrology. Real-time TV-rate stereo scanning electron microscopy has been available for many years and numerous papers using stereo microscopy have been published, especially in the biological sciences [7,8, 9,11]. Depth measurements of the stereo image has been applied to the metallurgical sciences. Lee and Russ [54] applied digital image processing, stereo-matching, and parallax measurements to measure surface height, slope, and wall angles [54], while Thong and Breton [130] applied the technique to three dimensional mapping of semiconductor devices. Kayaalp and Jain [47] investigated wafer pattern topography with a stereo SEM and, as described earlier, Postek et al [92, 93], demonstrated that when the electron beam modeling was compared to experimental data, wall slope information of gold absorber lines of an x-ray mask can be obtained from both the transmitted and backscattered electron images with a high degree of sensitivity. There is no reason why this facility cannot be developed and utilized further. One characteristic of the SEM is the large depth-of-field, but this is a variable, user-controllable parameter that can be manipulated to provide more data.

### 4.3 Development of SEM Standards

Currently, the need has been identified for three different standards for SEM metrology. The first need is for the accurate certification of the magnification of a nondestructive SEM metrology instrument (see Sec. 4.3.1); the second is for the determination of the instrument sharpness (see Sec. 4.3.2); and the third is an accurate linewidth measurement standard (see Sec 4.3.3).

#### 4.3.1 Magnification Certification

Currently, the only certified magnification standard available for the accurate calibration of the magnification of an SEM is NIST Standard Reference Material (SRM) 484. SRM 484 is composed of thin gold lines separated by layers of nickel providing a series of pitch structures ranging from nominally 1 to 50 μm. Newer versions have a 0.5 μm nominal minimum pitch. This standard is still very viable for many SEM applications. Certain limitations (e.g., size, low kV performance, etc.) presented by this standard for the particular needs of the semiconductor industry have been published previously [82] and NIST has been attempting to develop new standards designed to circumvent these limitations [94, 95]. During 1991–1992, an interlaboratory study was held using a prototype of the new low accelerating voltage SEM magnification standard. This standard, identified as NIST SRM 2090, is currently being fabricated.

##### Definition and Calibration of Magnification

**Fig. 12:**
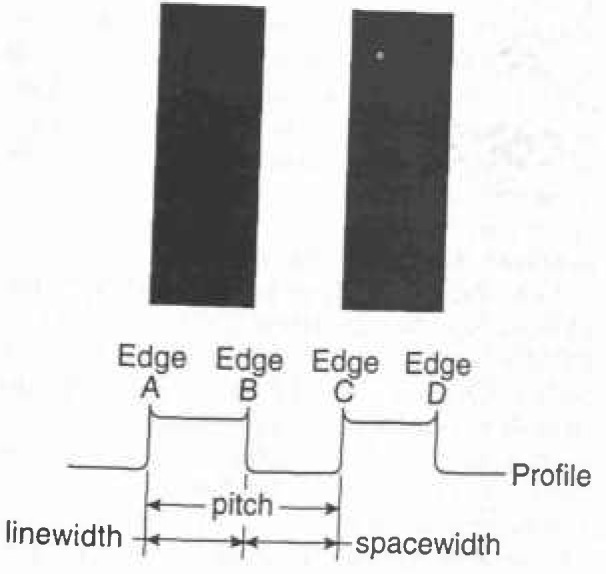
Graphic comparison between the measurement of pitch and width. Measurement of A to C or measurement of B to D defines the pitch of the sample. Measurement of A to B or C to D defines the linewidth of the sample and measurement of B to C defines the spacewidth.

**Fig. 13:**
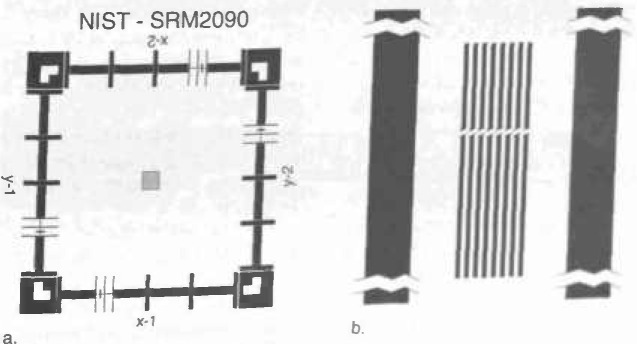
SRM 2090 artwork. (a) Low magnification of the 1 mm low magnification pattern; (b) higher magnification pattern showing eight of the nominal 0.1 μm pitch.

In typical scanning electron microscopy, the definition of magnification is essentially the ratio of the area scanned in both the X and Y directions on the specimen by the electron beam to that displayed in both the X and the Y directions on the photographic CRT. Because the size of the photographic CRT is fixed, by changing the size of the area scanned in both X and Y directions on the sample, the magnification is either increased or decreased. Today, where SEM metrology instruments are concerned, the goal is not only to calibrate the magnification as previously defined and discussed, but to calibrate the size of the pixels in both the X and the Y directions of the digital measurement system. For in these instruments, it is the measurement and not the micrograph that is important. Since, in most modern integrated metrology instruments, the digital storage and measurement system is common to the imaging, the “magnification” is also calibrated. It should be noted that because of the aspect ratio of the SEM display screen the number of pixels in X may differ from the number in Y, but the size of the pixel must be equal in both X and Y. This is an important concept, because in order for a sample to be measured correctly in both X and Y the pixel must be square. Such an X and Y measurement might be done on a structure such as a contact hole viewed normal (0° tilt) to the electron beam. The concept of pixel calibration and magnification calibration is essentially identical and pitch measurements can be used to adjust either [82,94, 95]. A pitch is the distance from the edge of one portion of the sample to a similar edge some distance away from that first edge (Fig. 12). Adjustment of the calibration of the magnification should not be done using a width measurement [38,39,82,94,95]. A pitch is the distance from the edge of one portion of the sample to a similar edge some distance away from that first edge (Fig. 12). Adjustment of the calibration of the magnification should not be done using a width measurement [38,39,82,94,95]. This is because width measurements are especially sensitive to electron beam/specimen interaction effects and other perturbing influences (see below and Sec. 4.1). This fact cannot be ignored or calibrated away especially if accurate SEM metrology is desired. Fortunately, it can be minimized by the use of a pitch type magnification calibration sample, such as SRM 484 [2], or the new SEM magnification calibration standard SRM 2090 [94, 95] when it is issued (Fig. 13). These standards are both based on the measurement of “pitch.” In a pitch standard, that distance is certified and it is to that certified value that the magnification calibration of the SEM is set. Under these conditions the beam scans a calibrated field width in both X and Y. That field width is then divided by the number of pixels making up the measurement system, thus defining the measurement unit or the pixel width. If we consider two lines separated by some distance, the measurement of the distance from the leading edge of the first line to the leading edge of the second line defines the pitch. Many systematic errors included in the measurement of the pitch are equal on both of the leading edges; these errors, including the effect of the specimen beam interaction, cancel. This form of measurement is therefore self-compensating. The major criterion for this to be a successful measurement is that the two edges measured must be similar in all ways. SEM pixel/magnification calibration can be easily and accurately calibrated to a pitch.

The measurement of a width of a line, as discussed earlier, is complicated in that many of the errors (vibration, electron beam interaction effects, etc.) are now additive. Therefore, errors from both edges are included in the measurement. The SEM magnification should not be calibrated to a width measurement since these errors vary from specimen to specimen due to the differing electron beam/sample interaction effects, as well as other factors [38,39,82,94,95]. Effectively, with this type of measurement, we do not know the accurate location of an edge in the video image; more importantly, it changes with instrument conditions. Postek et al. [94, 95], in an interlaboratory study of 35 laboratories, demonstrated that the width measurement of a 0.2 μm nominal linewidth varied substantially among the participants. Many factors contributed to this variation including instrument measurement conditions and measurement algorithms used [94, 95]. Calibration based on a width measurement requires the development of electron beam modeling, as described previously. This is the ultimate goal of the program at NIST and recently has been shown to be successful for special samples such as x-ray masks measured in the SEM (see below).

The dedicated “linewidth measurement” instruments or those with linewidth measurement computer systems often have an additional pixel calibration offset added to the magnification calibration in the software of the measurement function. This places a user defined “offset” or “correction” factor into the system. The measurement offset should be in *addition* to the magnification calibration and not in place of it. This offset can be determined from the measurement of an internal standard, NIST standard, or even the pitch of the actual device. Unfortunately, this offset does not usually affect the actual column scans or any of the above mentioned calibrations-only the “computer” measurement made directly with that system. Therefore, digital measurements made with the computer system may be relatively correct, but micrographs taken with that system may be out of (magnification) calibration by several percent. This software adjustment is really a *point calibration* in that it is usually done in the decade where the measurement is to be made. Erroneous results can also occur if the magnification is changed from that “calibrated” decade without rechecking the point calibration for that new decade.

##### Magnification Adjustment

The data obtained in the NIST interlaboratory study [94, 95] suggested that the method by which the magnification of the SEM is adjusted needs to be reengineered in many instruments. This is because the potentiometers used for setting the X and Y magnification calibration are often too insensitive or the calibration software step-size is too coarse for the repeatability required by today’s semiconductor industry needs. Such coarse adjustment was adequate for the older version of SRM 484 with its 1 μm nominal pitch, but for the new version of SRM 484 and the future SRM 2090, finer adjustment is needed. Adjustment sensitivity and procedures must also be the same in both the X and the Y directions. Today, with computer integration at all levels of the SEM electronics, this entire procedure could readily become automated.

#### 4.3.2 Sharpness Determination

**Fig. 14:**
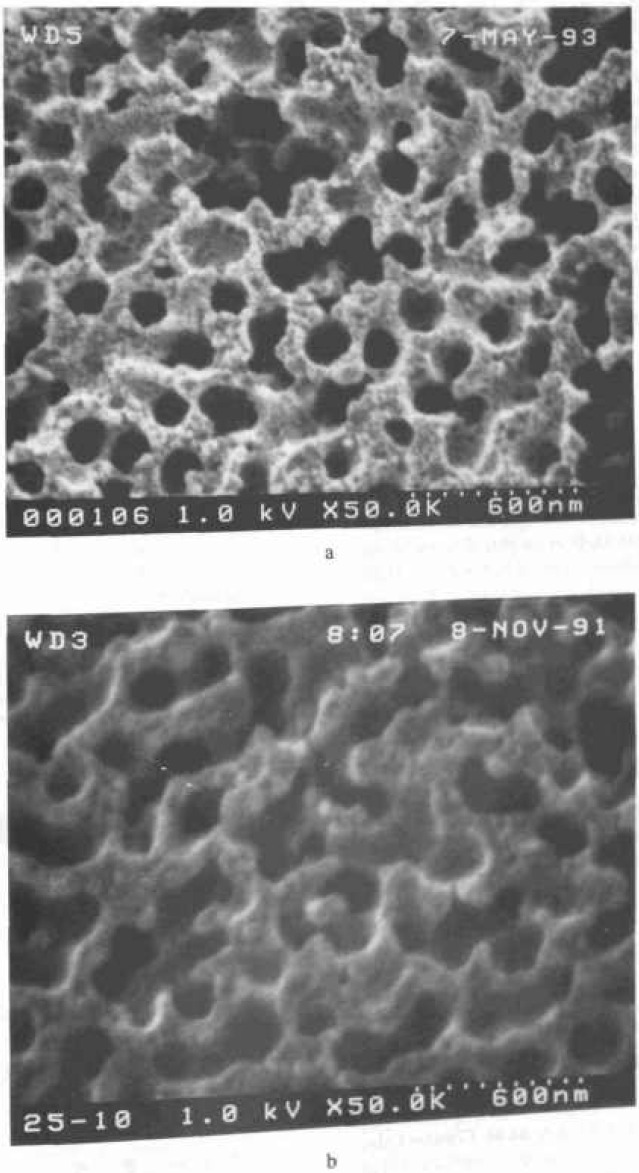
SEM sharpness *comparison using* a special etehed and coated biphasic glass specimen. (a) Instrument demonstrating good low accelerating voltage *resolution:* (b) instrument demonstrating poorer resolution.

The SEM resolution capabilities described in Table 2 are ideals. No SEM performs at that level continuously. If an SEM achieves that level of performance it degrades from that point with use. For example, apertures contaminate, alignments change, and electron source tips become blunted. All these factors (and many others) result in a loss of SEM performance. This performance loss may be a slow, gradual process as contamination builds up or may occur rapidly if a charged particle leaves the sample and is deposited in a sensitive location. Procedures for checking the performance level of the SEM need to become standardized and standard test samples need to be developed. Many of the basic criteria established for such a sample for use in an in-line instrument are similar to those described for the low accelerating voltage SEM magnification standard [82]. A sample developed for this type of work has been used successfully by NIST for the determination of the low accelerating voltage performance of laboratory SEM instrumentation (Fig. 3 and Fig. 14). This sample is based on the concept of the determination of sharpness and not “resolution.” Resolution determination implies a knowledge of the diameter of the electron beam. Whereas the concept of sharpness only requires an establishment of a sharpness criterion. The sharpness criterion can be determined visually or by computer using image analysis. The evaluation of samples similar to those used in Figs. 3 and 14 is currently in process for the establishment of this concept, as well as the development of a computer based analysis program. This sample is being designed to be readily applied to production instruments, as well as laboratory instruments.

##### Quality Micrographs vs Quality Measurements

Scanning electron microscopes evolved as picture taking instruments, and micrographs have historically been the final product. Modern scanning electron microscope metrological tools are data taking instruments and numbers are the final product. In many metrological instruments, the emphasis on the production of micrographs is minimized or even eliminated. However, both laboratory and in-line SEM instruments are similar in their general anatomy and design. The latter is generally more elaborately outfitted for rapid wafer transport, but, both operate on essentially the same principles and are subject to the same limitations. With the deemphasis of the recording of images, especially photographically, it is often felt that the image of the sample is less important than the numbers obtained. Yet, the only tie to the quality of the numbers obtained is the image or an analysis of the image. High quality image recording is primary to the quality of the data obtained and some “checks and balances” must be retained. Using the sample shown in Fig. 14, evaluation of the performance of the SEM can be visually determined from the micrographs or stored data. However, automated computer analysis is currently being investigated at NIST.

#### 4.3.3 Accurate Linewidth Standard

Accurate SEM linewidth standards are highly desired by the semiconductor industry. This industry is especially interested in standards for photoresist linewidth measurements. The knowledge of how to develop and measure an accurate linewidth standard for other materials such as masks used in x-ray lithography is already known and an accurate measurement has been accomplished [92, 93]. Building upon this knowledge, the generalized modeling necessary to develop other accurate linewidth-type standards may be able to be accomplished, as discussed above. But, until a flexible and accurate electron beam sample interaction model has been developed and tested, accurate linewidth standards cannot be issued.

### 4.4 Metrology of Contact Holes and Vias

**Fig. 15:**
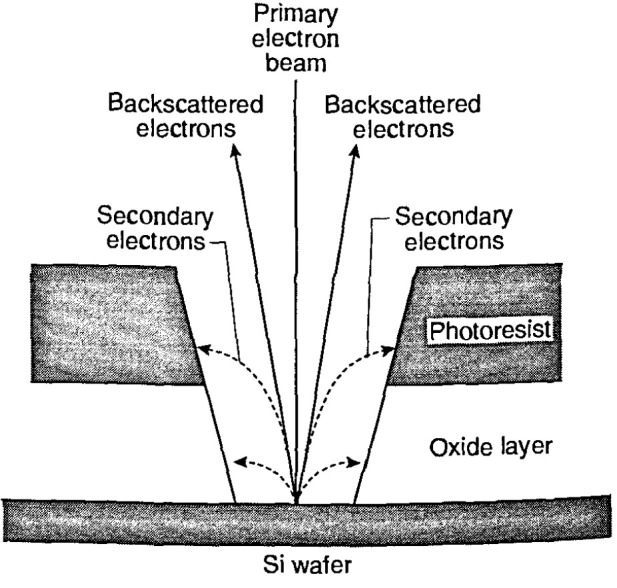
Drawing showing a contact hole and the problem with electron collection.

**Fig. 16:**
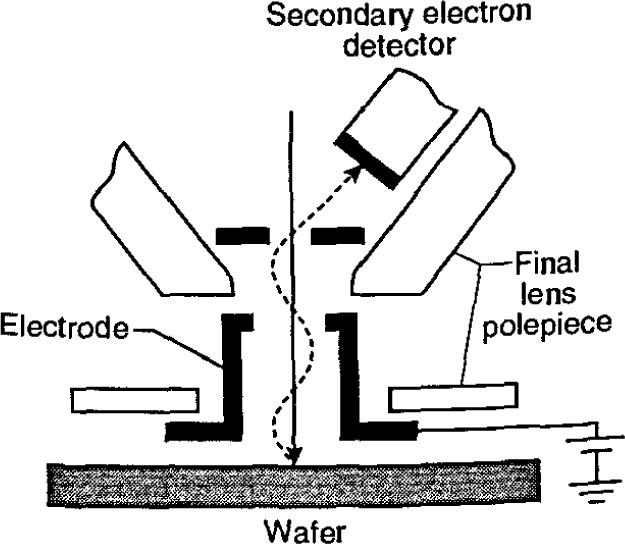
Drawing describing the field control concept (redrawn from Hitachi).

**Fig. 17:**
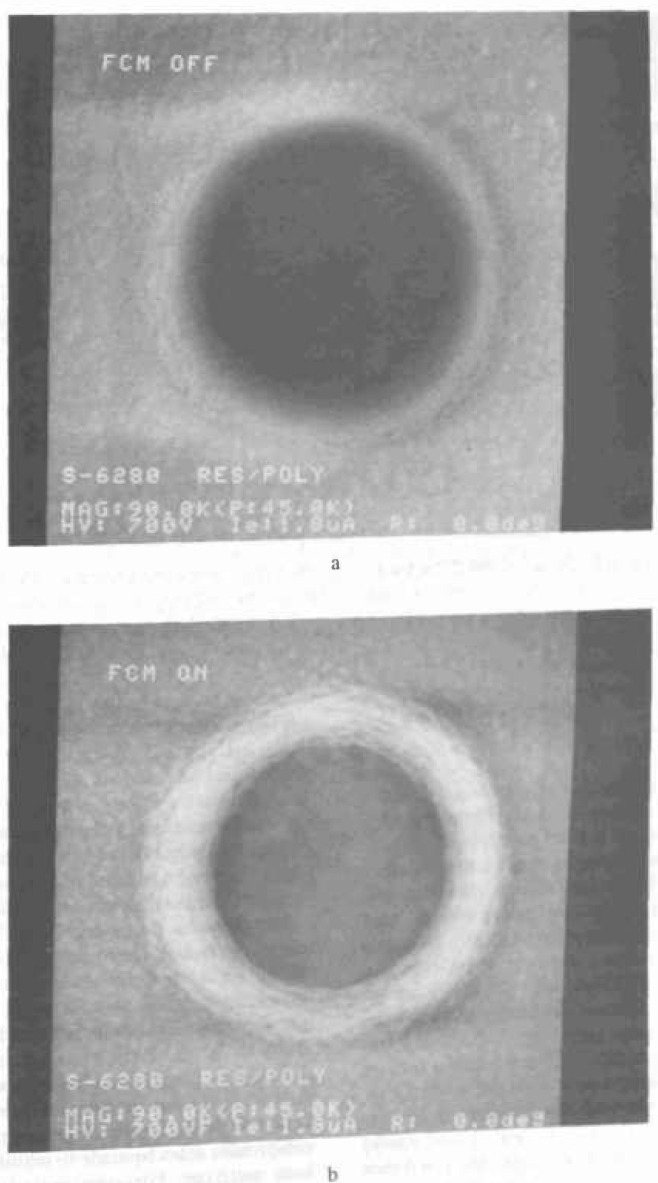
Enhancement of electron collection from a contact hole using the field control method. (a) Field control off; (b) field control on.

**Fig. 18:**
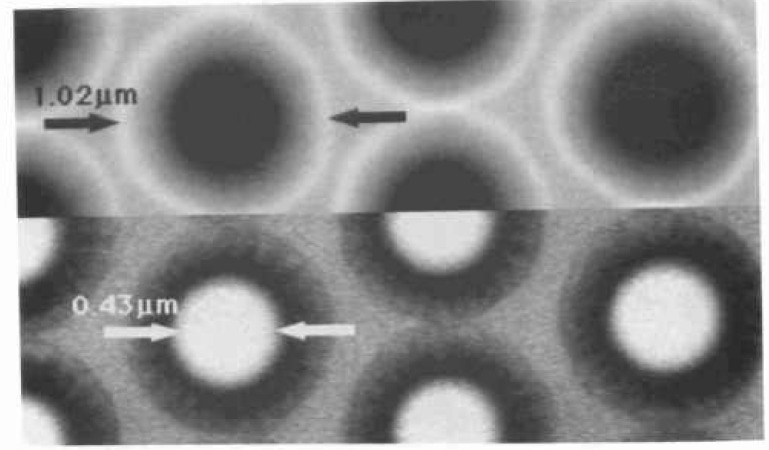
Micrographs showing contact holes viewed with dual microchannel-plate electron detectors. (a) Normal wide-angle collection; (b) high angle electron collection.

The metrology of contact holes and vias has become very important in recent years. It is important that contact holes and vias be inspected to see if they are properly etched and cleaned out and that they are fabricated in the proper dimensions. The inspection and metrology of contact holes has always presented a problem to SEM. Contact holes can be considered as being essentially small Faraday cups. Electrons entering the contact hole have a great difficulty leaving the hole again to be collected as a signal (Fig. 15). Workers have attempted to develop methods for looking into the contact holes. Postek et al. [91] demonstrated that by applying a positive or negative bias to a sample, the collection of secondary electrons from contact holes can be enhanced or reduced. Sample biasing is not easily implemented where large samples or wafer-transfer instruments are concerned; thus Hitachi (personal communication) has used a biasing technique referred to as “field control” to influence the collection of the electrons leaving the contact hole (Fig. 16). With the field control off, the contact hole has no detail (Fig. 17a) and with the field control, on detail becomes visible (Fig. 17b). Mizuno et al. [61] used high accelerating voltage to penetrate the photoresist in order to view the holes. Alternatively, Monahan et al. [66] have shown that the backscattered electron signal can be used to image the bottom of the contact holes (Fig. 18). This was accomplished by using two MCP backscattered electron detectors to collect the signal from the contact holes. The first detector, with a wide angle of collection, optimized the image from the top of the specimen while a narrow angle detector collected the image from the bottom of the hole.

### 4.5 Specimen Charging

Accumulation of charge on photoresist and other samples can result in nonreproducible and nonlinear measurement results. Therefore, the behavior of the total number of electrons emitted from a sample for each beam electron is extremely significant to nondestructive low accelerating voltage operation and metrology [65,83,85,86]. The two points where the total electron emission curve crosses unity (i.e., the E-1 and E-2 points) are the points where no net electrical charging of the sample is thought to occur [48]. During irradiation with the electron beam, an insulating sample such as photoresist or silicon dioxide can collect beam electrons and develop a negative charge causing a reduction in the primary electron beam energy incident on the sample. In principle, this could then also have a detrimental effect on the SEM magnification computation, as well as result in electron beam deflection. This charging can also have other detrimental effects on the primary electron beam and degrade the observed image. Backscattered electron collection has been successfully used to avoid the “obvious” charging effects on imaging and metrology using the secondary electrons. However, if charge build up is greater than a few electron volts, the backscattered electrons can also be affected. Few studies on charging at low accelerating voltage have been done and a great deal more work should be devoted to this issue.

**Fig. 19:**
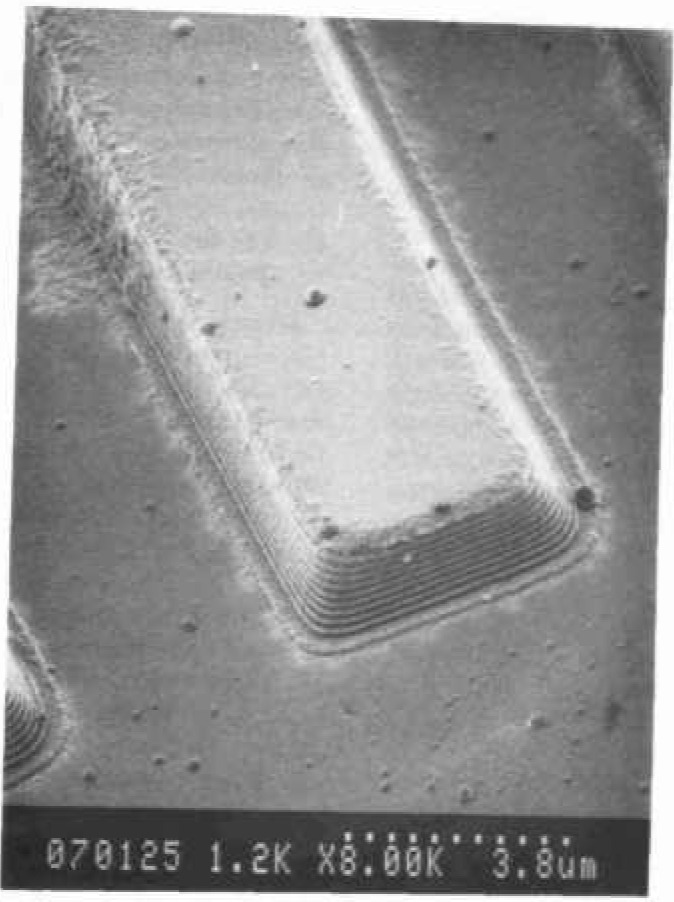
SEM micrograph of uncoated photoresist taken at 1.2 kcV accelerating voltage showing a tack of sample charging.

If the primary electron beam energy is chosen between E-l and E-2, there will be more electrons emitted than are incident in the incident beam, and the sample will charge positively. Positive charging is not as detrimental as negative charging, since this form of charging is thought to be only limited to a few electron volts because of the barrier it presents to the continued emission of the low energy secondary electrons. The reduction in the escape of the secondary electrons resulting from positive charging reduces signal as the secondary electrons are now lost to the detector. The closer the operating point is to the unity yield points E-1 and E-2, the less the charging effects. Each material component of a specimen being observed has its own total emitted electron/keV curve, and so it is possible that in order to completely eliminate sample charging, a compromise must be made to adjust the voltage for both materials. For most materials used in the present semiconductor processing, a beginning accelerating voltage in the neighborhood of 1.0 kV is sufficient to reduce charging and to minimize device damage (Fig. 19). It is clear that any accurate electron beam-specimen interaction model include the potential effects of sample charging.

Although operation at low beam energies is useful for the inspection of semiconductor samples with a minimum of sample damage and charging, a detrimental result is a reduction in the beam current available from the electron source (as compared with high voltage operation). The net result is that the signal-to-noise ratio is poorer. This leads to a loss in apparent sample detail. High brightness filaments and digital frame storage techniques for multiscan signal integration, or slow scan rates coupled with photographic or electronic integration, help to overcome this problem. The more abiding problem with low accelerating voltage operation is the lower resolution (as compared to the higher beam energy operation) characteristic of this mode of operation. It is also extremely important to continue to monitor the image sharpness to ensure that the instrument performance is up to specification (see Sec. 4.3.2).

#### 4.5.1 Environmental SEM

Specimen charging can be dissipated at poor vacuum pressure “Environmental” scanning electron microscopes have been introduced in several areas of general SEM applications in order to look at samples generally prone to charging. Low chamber vacuum for semiconductor processing has two consequences: the first is on throughput and the second is on specimen charging. For many years, scanning electron microscopy has routinely been done at relatively high vacuum in the specimen chamber. For metro-logical applications, this initially posed a complication because of the reduction in throughput that the pumpdown of the wafers from atmospheric pressure to the working chamber pressure posed. One solution developed was to cache wafers in a prepumping chamber, then move them individually into the specimen chamber when needed for viewing. The alternative is to view the wafers in poor vacuum in a specialized environmental SEM metrology instrument. Environmental SEM is relatively new to the overall SEM field and a great deal of work is being done to understand the mechanisms of operation. The reader is directed to the work of Danilatos for further information [20, 21].

### 4.6 Universal Measurement Algorithm for Comparison

**Fig. 20:**
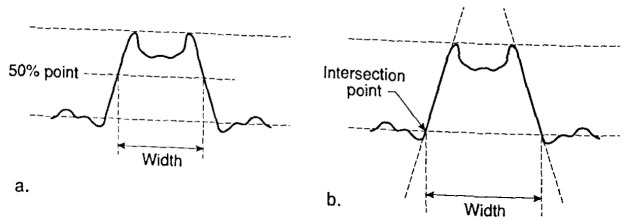
Diagrammatic comparison of the difference between two common measurement algorithms on the reported “width” measurement. (a) Threshold crossing algorithm; (b) linear regression algorithm. Please note the didderence in meassument result; possible between the two algorithms.

Each metrological SEM has had developed for it a set of measurement algorithms. These algorithms are commonly manufacturer and sometimes instrument specific. Many of these algorithms are based upon instrumental convenience or in some cases experimental observation [60, 151]. The matching of data from various instruments is desirable [98], but often difficult to undertake where instruments from different manufacturers are concerned. Where a pitch measurement is concerned, the type of measurement algorithm employed is not as important because of the self compensating nature of that measurement. However, when a linewidth measurement is employed, the measurement is not self-compensating and errors are additive. In this case, the choice of algorithm becomes extremely important [94, 95]. Different samples may also require different data analysis techniques [106, 107]. No measurement algorithm based upon accurate electron beam modeling currently exists. Therefore, none exists in commercial instrumentation. However, the x-ray mask modeling results (described earlier) could lead to one [92, 93]. For instrument testing and comparison purposes a common algorithm and data handling techniques should be adopted. This would include known data processing (smoothing, etc.) and measurement procedures. *Raw (unprocessed) data should always be able to be obtained from a measurement system.* This will readily permit the comparison of the experimental data to modeled data. A common algorithm should also be transportable and capable of being used to compare instruments. The need for this was clearly pointed out in the SEM interlaboratory study [94, 95], where data from several different types of instruments were compared. In that study, no viable comparison of linewidth measurement data could be obtained because of the differences between handling of the data in the various instruments and the algorithms involved (Fig. 20).

### 4.7 Universal Measurement Data Storage and Transmittal

Measurement data and ¡mages need to be transported throughout the laboratory and the wafer fabrication facility. Instruments of many different manufacturers may be used for different purposes. In some cases different models of instruments from the same manufacturer cannot even communicate with each other. This problem was clearly pointed out during the SEM interlaboratory study [94, 95], when data supplied to NIST on disk could not be used. Issues regarding compatibility with existing software, accurate data representation, data compactness on disk, and rates of data transfer have been raised and standard formats suggested [23]. A common data transfer and storage format should be established and adopted for *all* metrological instruments and adhered to so that image files or data can be easily transported from any instrument to personal computers and back again, as needed.

### 4.8 Lens Hysteresis/Compensation Correction

Many in-line scanning electron microscopes always operate under the same operating parameters day-after-day. Other instruments operate through a range of instrumental conditions. The electromagnetic lenses comprising the column of the SEM may exhibit the effects of lens hysteresis following changes in instrument operating conditions (i.e., especially during accelerating voltage changes) [86]. This limitation on metrology became quite apparent in many instruments during the SEM interlaboratory study [94, 95]. Many metrological instruments have some form of lens hysteresis/compensation correction, but some do not. This capability should be checked by each user of the instrument to determine if it is present in their instrument and that it functions properly.

### 4.9 Specimen Contamination/Specimen Damage

The effects of the electron beam on the sample can be two-fold: first, the beam can generate specimen contamination from its interaction with hydrocarbons, either in the specimen surface or from the instrument; second, it can induce damage to the actual devices.

#### 4.9.1 Contamination

Sample contamination is inevitable in all but the fully dry-pumped SEM inline instruments. Contamination results from sample handling, the environment, and the instrument. Hydrocarbons interact with the electron beam and form a layer on the surface. The speed at which this deposition occurs varies with the amount of hydrocarbon (or other contaminant) available to interact, as well as the operational conditions of the instrument (i.e., beam current) and electron beam dwelltime on the sample [27,35,36]. Dry nitrogen purging and backfilling is helpful in reducing contamination. Subsequent post processing with oxygen plasma can often clean off this contamination from the wafer [94, 95].

#### 4.9.2 Damage

Irradiation damage of some devices viewed in an SEM at higher accelerating voltages has been reported [102, 131]. Tocci et al. [102] found damage in MOSFET devices irradiated at 2 keV accelerating voltage. Erasmus [24] observed that photoresist can change dimension under electron beam inspection. However, it was also found by that author that an optimum dose can be identified where no damage occurs. Both Van Asselt [138] and Robb [105] detected no damage when the accelerating voltage was restricted to below 3 kV and the inspection was followed by a high temperature anneal. Bhattacharya [6] reported no significant radiation-induced gate insulator damage with an exposure of 1 keV electrons. It was further concluded by these authors that SEM examination of finished devices could be accomplished with no radiation damage below 7 keV accelerating voltage. It is currently felt that it is safe to inspect most devices in an SEM during production.

### 4.10 Reduced Sensitivity to External Nolse

The SEM metrology tool is expected to perform in a relatively “hostile” environment [64, 86], Vibration from numerous sources and stray fields are quite common in the wafer fabrication facility. Some field emission instruments are exceptionally sensitive to stray electromagnetic fields. In some instances of high field intensity, mu-metal enclosures had to be built to completely surround the SEM column. In other instances the instrument itself has been found to be the source of the perturbing field. Regardless of the source, these instruments must be designed better to better handle the “hostile” environment in which they work.

## 5. Conclusions

Scanning electron microscope metrology is a dynamically changing field. The needs of the semiconductor industry have driven numerous improvements in the instrumentation. These improvements have been felt throughout the SEM field and have enabled a great deal of progress to be made in all applications of this instrument. In this paper, several areas of misconception regarding instrument performance and capabilities have been clarified. Clearly, further effort is needed in fundamental areas of scanning electron microscopy metrology. Many of these areas will be dealt with in future years resulting in even further improved instrumentation.

## Figures and Tables

**Table 1:** Typical scanning electron microscope metrology instrument specifications

Minimum predicted feature size	0.1 μm
Image resolution (@ 1.0 kV)	<8.0 nm
Accelerating voltage range	General purpose 0.5 kV to 30 kV In-Line 0.5 kV to ~2.5 kV
Magnification	100 × to 300 000 ×
Wafer size capabilities (in mm)	100, 125, 150, 200
Cleanliness	<2 particles added/pass
Mean time between failure	>1500 h
Availability	>95 %
3*S* Repeatability (lines and spaces)	Static <5 nm Dynamic <10 nm
3*S* Repeatability (contact holes and vias)	Static <10 nm Dynamic <20 nm
Wafer throughput	>20/h
Stage speed	>50 mm/s
Pattern recognition—probability of detection	>99 %
Pattern recognition—position uncertainty	±0.2 μm

**Table 2:** SEM achievable resolution

Detector position	Accelerating voltage	tn-Lens FE	Extended field FE	Post-Lens FE	Post-Lens LaB_6_	Post-Lens tungslen
Upper	30.0 kV	0.7 nm	1.5 nm			
	1.0 kV	3.5 nm	4.5 nm			
Lower	30.0 kV			1.5 nm	2.5 nm	3.5 nm
	1.0 kV			5.0 nm	7.5 nm	10.0 nm

**Table 3:** Comparison of pertinent electron source characteristics

	Unit	Tungsten filament	LaB_6_ filament	CeB_6_ filament	Cold field emission filament	Schottky field emission filament
Reference number		99	14, 76	12	75,99,134 136, 143	75,99,125 126, 134 135, 136 139
Angular current intensity	mA/sr	n/a	n/a	n/a	<0.1	0.1 to 1.0
Source brightness	A/(cm^2^·sr)	10^6^	10^7^	10^7^	10^9^	10^8^ to 10^9^
Emitting surface area	μm^2^	>>1	>1	>1	0.02	0.2
Crossover or virtual source diameter	nm	>10^4^	>10^3^	>10^3^	3 to 5	15 to 25
Energy spread	eV	1 to 3	1 to 1.5	1 to 1.5	0.2 to 0.3	0.3 to 1.0
Source temperature	K	2500 to 2900	1800	1800	300	2.8
Work function	eV	4.5	2.6	2.4	4.5	2.8
Operating vacuum	Pa	10^−4^	10^−6^	10^−6^	10^−9^ to 10^−11^	10^−6^ to 10^−9^
RMS short term beam current stability	*%*	<1	<1	<1	4 to 6	<1
Typical service life	h	40 to 100	1000	>1500	>2000	>2000

## Footnotes

1 The term repeatability is used in this paper in place of the more commonly used term by the semiconductor industry-precision. This is beeausc NIST has adopted the ISO definitions for metrology and its approach to measurement uncertainty [153, 154].

2 Certain commercial equipment, instruments, or materials are identified in this paper to foster understanding. Such identification does not imply recommendation or endorsement by the National Institute of Standards and Technology, nor does it imply that the materials or equipment identified are neecssarily the best available for the purpose.
